# Are the stages of change relevant for the development and implementation of a web-based tailored alcohol intervention? A cross-sectional study

**DOI:** 10.1186/1471-2458-12-360

**Published:** 2012-05-17

**Authors:** Daniela N Schulz, Stef PJ Kremers, Hein de Vries

**Affiliations:** 1 Department of Health Promotion, Maastricht University/CAPHRI School for Public Health and Primary Care, Faculty of Health, Medicine and Life Sciences, P.O. Box 616, 6200, MD, Maastricht, the Netherlands; 2 Department of Health Promotion, Maastricht University/Nutrition and Toxicology Research Institute Maastricht (NUTRIM), Faculty of Health, Medicine and Life Sciences, P.O. Box 616, 6200, MD, Maastricht, the Netherlands

## Abstract

**Background:**

Computer-tailored programs are a promising tool to stimulate health behavior change, such as reducing alcohol intake. Yet more research is needed to assess whether groups differing in their motivational level to change may need different types of feedback. Furthermore, it is unknown whether motivational level may also determine reactions to computer-tailored interventions. Our aim is to identify the potential relevance of the application of the stages of change concept in the development and implementation of alcohol interventions.

**Methods:**

A web-based instrument was used to disseminate a questionnaire and to provide tailored feedback messages among adults in the Netherlands (N = 170; 96 females). Motivational level was assessed by the stage of change construct. The survey furthermore assessed alcohol consumption, attitude, social influence, self-efficacy, and program evaluation (i.e., survey items, tailored advice, layout and functionality of the program). The Least Significant Difference method was used to compare people in different stages of change with regard to psychosocial determinants of drinking behavior and program evaluation.

**Results:**

Of the respondents, 34.1% (n = 58) reported no intention to change to healthier drinking habits in the foreseeable future (precontemplation), 22.9% (n = 39) intended to improve their drinking behavior in the near future (contemplation/preparation) and 42.9% (n = 73) reported to currently adhere to the Dutch alcohol consumption guidelines (action/maintenance). When comparing the three groups, people in the action or maintenance stage reported the lowest number of pros of drinking alcohol, having most healthy drinking role models and the highest levels of self-efficacy regarding healthy drinking in difficult situations, whereas precontemplators reported to receive the least social support regarding healthy drinking. In general, the intervention was positively evaluated, but it seemed to be most appreciated by contemplators and preparers.

**Conclusions:**

Stage-matched interventions may be useful to encourage people to reduce their alcohol intake. Different factors seem to be important for people in different motivational stages. Longitudinal studies are needed to determine whether these factors also predict stage transition. The intervention could be optimized by tailoring the feedback messages more precisely to the needs of people in different motivational stages, for example by applying the different processes of change.

## Background

Heavy alcohol consumption often leads to multiple negative consequences, such as physical [1-4], mental [5,6], social [7] and economic problems [8-10]. It is one of the most important risk factors for chronic diseases, like cardiovascular diseases and cancer [7,11], and mortality [12]. Unhealthy alcohol consumption, defined as the tendency to drink more than two (females) or three (males) standard drinks per day [13-15], is widely prevalent [16-18]. In 2007, around 14.0% of Dutch men and 10.5% of Dutch women failed to comply with the country's alcohol guidelines [19]. Other drinking patterns, like binge drinking, also seem to have an impact on various health-related problems and are widely common as well, especially among youngsters and young adults [12,20]. The high prevalence of unhealthy drinkers indicates the need for interventions to encourage people to reduce their alcohol intake.

Research has shown that computer tailored interventions, in which information is adapted to the characteristics and needs of the individual in order to give more personal and relevant advice [21], are a promising tool to stimulate behavior change as well as to maintain healthy behaviors and to prevent relapse. Computer-tailored programs show key benefits in comparison with non-tailored materials, e.g. they contain less unnecessary and more attractive and more relevant information [22,23], they are cost-effective [24], they seem to be more effective in behavioral change [25], and the tailored messages are more often read, saved, printed out, remembered and discussed with others [21,26-28].

**Figure 1:**
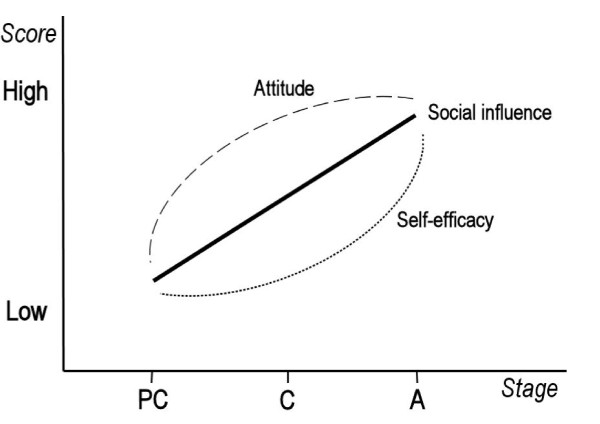
**The Ø–pattern (De Vries & Backbier, 1994)**. A graphical representation of a stage model, including attitude, social influence and self-efficacy (PC = precontemplation, C = contemplation, A = action).

Computer-tailored interventions are frequently based on theories [e.g. [29,30]. One potential framework for the development of such an intervention is the Transtheoretical Model (TTM) [31,32]. The TTM distinguishes five stages of change: *precontemplation*; *contemplation*; *preparation*; *action*; and *maintenance*. The TTM describes the likelihood that people in different stages may require different intervention strategies in order to move onto a further stage [33,34]. This means that the tailored feedback messages should be tailored to the stage of change of the individuals in order to motivate them to adopt or maintain healthy behaviors. Despite several criticisms [35-40], various studies found that certain factors may be more relevant in certain stages than others [41,42]. According to De Vries and Backbier [43], people in different motivational stages differ in terms of attitude, social influence and self-efficacy, as presented in Figure1. The pattern has been confirmed for smoking cessation by other studies [41,43,44], but not yet for alcohol consumption.

Figure1 illustrates that changing attitudes is a crucial first step in a behavioral change process. When comparing people in the different stages of change, precontemplators are assumed to perceive more pros than cons of the “unhealthy” behavior. In the contemplation stage, the pros and cons are more in balance whereas in the action stage people experience more cons than pros. This crossover is a typical phenomenon that distinguishes precontemplators from contemplators and preparers [45]. With regard to social influence, the impact of this factor increases gradually across the behavioral change process. Precontemplators and contemplators are assumed, however, not to differ in levels of self-efficacy. They do, though, have lower levels of self-efficacy than people in the preparation and action stage. This principle, referred to as the Ø–pattern by De Vries and Backbier [43], can also be used to guide the development of stage-matched interventions [41]. The first goal of this paper is to study whether a similar pattern can also be observed for the intention to reduce alcohol intake in Dutch adults.

Another important issue concerning computer tailoring is whether tailored programs are appreciated as much by respondents in all stages of change. On the one hand, one may argue that the stage-matched feature of these types of interventions may lead to as much appreciation from respondents in precontemplation as those in preparation, because optimally respondents of a tailored approach receive no redundant or irrelevant information. Yet, because respondents in precontemplation are not yet ready to think about changing their behavior, it is therefore likely that – despite the tailored approach – respondents will be less favorable in terms of their evaluations than those in preparation. The second goal of this paper is therefore to assess potential differences in appreciation between respondents in different stages.

This study assesses the factors associated with intention to adhere to Dutch alcohol consumption guidelines based on the TTM. The first goal of our study is to compare adults of the general population in different stages of change with regard to the following psychosocial predictors of drinking: (1) attitude; (2) social influence; and (3) self-efficacy. The second goal of this study is to investigate if the appreciation of the computer-tailored alcohol program is related to the motivational stage of a person. Our aim is to identify the potential relevance of possible stage-matched interventions regarding Dutch alcohol consumption guidelines.

## Methods

### Sampling procedure and design

This study is part of a large trial assessing the effects of adopting web-based computer tailoring on lifestyle behaviors and was approved by the Medical Ethics Committee of Maastricht University and the University Hospital Maastricht (NL27235.068.09/MEC 09-3-016).

Our present research was a cross-sectional, quantitative web-based questionnaire study, including written feedback messages. In spring 2008, the recruitment of respondents took place through advertisements and articles in different local newspapers (free sheets) in the Limburg province, which is located in the southern part of the Netherlands, and the distribution of flyers and posters in public places, such as supermarkets, universities, hospitals and airports, in both rural and urban areas. All people with a command of the Dutch language, at least 18 years old and with access to the Internet were invited to participate in this study. The purpose of the study was explained to the respondents and confidentiality was assured. Respondents did not receive any incentives for participation.

In total, 230 people visited our website. Of the 212 individuals (92.2%) who gave informed consent and started to fill out the questionnaire, 170 respondents (80.2%) completed the main part containing items about attitude, social influence, self-efficacy and stages of change, and were included in the analyses. The program was evaluated by 146 respondents (85.9%).

### The intervention

To deliver personalized advice, three inter-related elements were necessary: 1. a *screening instrument* (questionnaire) to measure alcohol consumption and influencing determinants; 2. a *message source file* containing all tailoring messages; and 3. a *computer program* that offered the opportunity to analyze the screening results and to select the correct messages from the message file [21,46]. A web-based instrument was used to create and conduct an online questionnaire and to design and implement the tailored advice. By means of tailored formulas responses to questions yielded the tailored advice that respondents received on their screen and which could also be printed as a pdf file.

### Questionnaire content

A questionnaire, based on earlier computer tailored studies [27], was used to measure demographics, alcohol consumption, attitude, social influence, self-efficacy and stages of change as well as to evaluate the computer-tailored program, particularly the tailored feedback messages.

#### Demographic information

The sample group was asked to provide the following demographic characteristics: gender, age, nationality, level of education (“no education, primary school, vocational school, secondary vocational school or high school,” i.e. low level of education; or “higher vocational school or university,” i.e. high level of education), relationship status and number of children.

#### Alcohol consumption

Alcohol consumption was measured by using the five-item Dutch Quantity-Frequency-Variability (QFV) questionnaire [47]. Respondents could choose the type of alcoholic drinks that they consumed during the last twelve months, such as beer, wine, cocktails and sherry. Respondents were asked to indicate how many working days (Monday to Thursday) they had consumed alcohol during the past twelve months. Additionally, they were asked to indicate the number of standard drinks (consisting of approximately 10 g of pure alcohol) they usually consumed on these occasions. Similar questions were asked concerning alcohol consumption during weekend days (Friday to Sunday).

#### Attitude

To measure attitude concerning alcohol, participants had to indicate on a five-point scale to what extent they disagreed (−2) or agreed (+2) with 45 given statements. Principal component analyses were performed in order to identify factors. The factor analysis, using direct oblimin rotation, revealed two components of attitude: advantages (pros) and disadvantages (cons) of drinking [see Additional file 1]. Pros were assessed by 21 items (α = 0.93), such as “When I drink alcohol, I feel happier.” The cons of drinking were assessed by another 24 items (α = 0.95), such as “When I drink alcohol, the risk of developing cancer increases.”

#### Social influence

Social influence was measured by eight items with a five-point scale, ranging from “totally disagree” (−2) to “totally agree” (+2). By means of factor analysis, two components of social influence were found: modeling and support [see Additional file 1]. *Modeling* was measured by asking participants whether their partner, family, friends and colleagues met the guidelines for adequate alcohol intake. Four statements were given (α = 0.70), such as “My partner does not drink more than three glasses of alcohol per day.” *Social support* was assessed by asking participants whether they were encouraged by their partner, family, friends and colleagues to meet the alcohol guideline. Four statements were given (α = 0.85), such as “My partner encourages me not to drink more than two glasses of alcohol per day.”

#### Self-efficacy

To measure self-efficacy towards healthy drinking, participants had to indicate on a five-point scale whether they felt “certainly unable” (−2) or “certainly able” (+2) to drink in a healthy manner in 22 different situations. Factor analysis revealed three components: social self-efficacy, emotional self-efficacy and routine self-efficacy [see Additional file 1]. *Social self-efficacy* was assessed by using seven items (α = 0.94), such as “I am able to drink no more than two glasses of alcohol when I am at a party.” *Emotional self-efficacy* was assessed by using 10 items (α = 0.97), such as “I am able to drink no more than two glasses of alcohol when I am sad.” *Routine self-efficacy* was assessed by using five items (α = 0.88), such as “I am able to drink no more than two glasses of alcohol after doing sports.”

#### Stages of change

Six statements were formulated to classify each individual in one of the stages of change regarding healthy drinking (feedback concerning the appropriate consumption was tailored to the respondent’s gender): “I do not plan to drink a maximum of two/three glasses of alcohol per day”, which means that respondents did not intend to adhere to the alcohol guideline in the future, and “I plan to drink a maximum of two/three glasses of alcohol per day, but not within the next six months” (PC), “I plan to drink a maximum of two/three glasses of alcohol per day within the next six months” (C), “I plan to drink a maximum of two/three glasses of alcohol per day within the next month” (P), “I already drink a maximum of two/three glasses of alcohol per day, but started during the last six months” (A) and “I have drunk a maximum of two/three glasses of alcohol per day for more than six months” (M). The last two items were meant for people who already adhere to the alcohol guideline for a while, including nondrinkers (for less than six months and for longer than six months, respectively). Respondents had to choose the most applicable statement.

#### Program evaluation

The *items of the questionnaire* were evaluated by five questions, such as “The questions were easy to answer,” with a five-point scale (totally disagree (1)-totally agree (5); α = 0.67). The tailored *pieces of advice* were evaluated by 17 questions, such as “In my opinion, the advice was relevant,” with a five-point scale (totally disagree (1)-totally agree (5); α = 0.87). Moreover, respondents were asked to rate the tailored advice on a scale from one (bad) to 10 (excellent). The *layout and the functionality* of the Internet program were evaluated by seven questions, such as “I like the layout of the website” and “I think the program is user-friendly,” with a five-point scale (totally disagree (1)-totally agree (5); α = 0.72). Additionally, respondents were asked to rate the whole website on a scale from one (bad) to 10 (excellent).

### Tailored messages

The structure of the program was based on other computer-tailored programs which have already been proven to be effective in changing lifestyle behaviors, such as in increasing smoking cessation [30] and in increasing amounts of physical activity [48]. The I-Change model [49] was used as the theoretical framework for the tailored advice. The message file was used to generate tailored messages about adhering to the guideline for adequate alcohol intake, alcohol consumption, pros and cons of alcohol consumption, social influence and self-efficacy. Text-based messages were given in combination with suitable pictures. For example, we included tips and suggestions about how to decrease one's alcohol intake. In case respondents reported to drink in a healthy manner, maintenance-feedback was given which means that people were stimulated to also drink in a healthy manner in the future. With respect to social influence, feedback was given on how to deal with the presence of drinking others, like friends or family. Furthermore, we gave special personalized advice about social, emotional and routine risk situations. Graphic feedback was also used: A diagram was made which showed the relationship between the advantages and disadvantages of alcohol intake as experienced by the participants.

### Statistical analyses

The data were analyzed with the statistical program SPSS, version 15.0. Descriptive statistics were used to describe the participants’ characteristics and their alcohol intake. Different groups (i.e. based on their actual stage of change) were compared with respect to demographics and drinking behavior by use of Chi-square tests for discrete variables and *F*-tests for continuous variables. In the case of significant main effects, variables were included in the analyses as covariates. Next, the groups were compared with regard to psychosocial determinants of drinking behavior and their program evaluation. Standardized *T*-scores were calculated with a mean of 50 and a standard deviation of 10. Differences between the groups were determined with the “Least Significant Difference” (LSD) method. For all tests, the significant value was set at *p* < .05.

## Results

### Participants’ demographics and drinking behavior

Participants were aged from 18 to 74 years, with a mean age of 44.62 years (SD = 14.52). Slightly more women (n = 96; 56.5%) than men (n = 74; 43.5%) took part in this study. Nearly all respondents were of Dutch origin (n = 155; 91.2%). Two-thirds of the participants were classified as highly educated (n = 104; 61.2%). The majority were in a relationship (n = 123; 72.4%) and one quarter were living together with their children (n = 45; 26.5%).

The mean daily alcohol intake among the whole sample was 2.69 standard drinks (SD = 3.04), with a statistically significant difference for males and females [*t*(107.93) = 4.98; *p* < .001]. Men had a mean daily alcohol intake of 4.00 glasses (SD = 3.60), whereas women had a mean daily alcohol intake of 1.68 glasses (SD = 2.02). Sixty-six respondents (38.8%) failed to meet the alcohol guideline.

### Stages of change

When the 170 respondents were grouped according to motivation to change, 34.1% did not intend to change to healthier drinking habits within the next six months (PC: n = 58), 22.9% intended to drink in a healthy manner at some time in the near future (C: n = 15; P: n = 24), 4.1% had started to drink in a healthy manner during the past six months (A: n = 7) and 38.8% had already been drinking in a healthy manner for more than six months (M: n = 66). Because of the distribution of respondents among the different stages of change, three groups were formed. Precontemplators remained as one group (PC), contemplators and preparers were combined into one group (CP) and defined as those planning to reduce their alcohol intake in the near future and those in the action and maintenance stages were grouped together and defined as those currently adhering to the guideline (AM).

The distribution of respondents over the three groups differed significantly for gender, age and drinking behavior (Table1). Contemplators/preparers drank more alcohol than precontemplators. Females were more likely to be in the action or maintenance stage than males. Contemplators/preparers were significantly older in comparison with the other two groups. Owing to these differences, the analyses were corrected for gender and age.

**Table 1:** Demographics and drinking behavior variables of the sample group (N = 170)

**Variables**	**Precontemplation (PC; n = 58)**	**Contemplation/Preparation (CP; n = 39)**	**Action/Maintenance (AM; n = 73)**	** *χ* **^**2**^	**F**	**Sign.**
**Gender**				9.54		.008
*Female*	28 (48.28%)	17 (43.59%)	51 (69.86%)			
*Male*	30 (51.72%)	22 (56.41%)	22 (30.14%)			
**Age**					10.31	.000
*Years (mean [SD])*	41.14 [15.11]	53.44 [10.59]	42.38 [14.86]			
**Nationality**				n/a		n/a
*Dutch*	54 (93.10%)	37 (94.87%)	64 (87.67%)			
*Other*	4 (6.90%)	2 (5.13%)	9 (12.33%)			
**Education**				.79		n.s.
*Low*	22 (38.60%)	16 (42.11%)	24 (33.80%)			
*High*	35 (61.40%)	22 (57.89%)	47 (66.20%)			
**Relationship status**				1.46		n.s.
*Single*	19 (32.76%)	11 (28.21%)	17 (23.29%)			
*In relationship*	39 (67.24%)	28 (71.79%)	56 (76.71%)			
**Children**				.44		n.s.
*Yes*	15 (25.86%)	9 (23.08%)	21 (28.77%)			
*No*	43 (74.14%)	30 (76.92%)	52 (71.23%)			
**Quantity**					17.28	.000
*Drinks per day (mean [SD])*	3.13 [3.45]	4.51 [3.08]	1.37 [1.89]			
**Frequency**					32.05	.000
*Number of days (mean [SD])*	3.28 [2.21]	6.19 [1.30]	3.13 [2.24]			
**Guideline**				32.05		.000
*Compliance*	32 (55.17%)	8 (20.51%)	64 (87.67%)			
*Non-compliance*	26 (44.83%)	31 (79.49%)	9 (12.33%)			

### Cognitive determinants of drinking behavior

**Figure 2:**
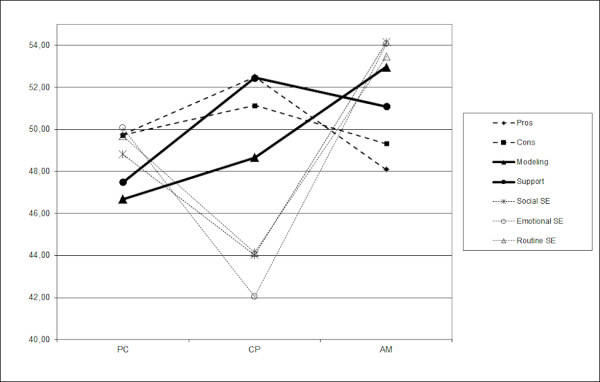
**Standardized *T* -score patterns of attitude (pros, cons), social influence (modeling, support) and self-efficacy (social, emotional, routine) in the stage groups**. PC = precontemplation; CP = contemplation/preparation; AM = action/maintenance.

To compare the three groups in terms of cognitive determinants, Figure2 represents the standardized *T*-score patterns on pros and cons, social modeling and support and social, emotional and routine self-efficacy. Table2 shows the standardized *T*-scores and the LSD contrasts in order to identify potential differences between the stages of change.

**Table 2:** **Standardized *T* -scores of attitude (pros, cons), social influence (modeling, support) and self-efficacy (social, emotional, routine) in the stage groups**

	**PC; n = 58**	**CP; n = 39**	**AM; n = 73**	**LSD contrasts**
**Pros**	49.76	52.50	48.09	PC, CP; PC, AM; CP > AM
**Cons**	49.73	51.14	49.32	PC, CP, AM
**Social modeling**	46.69	48.66	52.97	PC, CP < AM
**Social support**	47.49	52.45	51.09	PC < CP, AM
**Social self-efficacy**	48.83	44.03	54.14	PC > CP < AM; PC < AM
**Emotional self-efficacy**	50.09	42.05	54.05	PC > CP < AM; PC < AM
**Routine self-efficacy**	49.70	44.15	53.45	PC > CP < AM; PC < AM

*Note.* PC = precontemplation; CP = contemplation/preparation; AM = action/maintenance.

CP < AM: the mean score of CP is significantly lower than that of AM.

PC, CP, AM: the mean scores of PC, CP and AM are equal.

With regard to attitude, respondents in CP scored significantly higher on the pros than people in AM. The scores for cons did not differ significantly between the three groups. People in AM scored significantly higher on social modeling than those in PC and CP. Respondents in PC reported perceiving less social support than those in CP and AM. Respondents in PC and CP showed lower levels of self-efficacy in social, emotional and routine situations compared with those in AM whereas PC showed higher levels than CP.

### Differences in program evaluation

Table3 represents the standardized T-scores and the LSD contrasts used to identify potential differences in the program evaluation data between the three groups. Respondents in CP reported more often than people in PC and AM that the advice would help to stimulate them to change their drinking behavior. Moreover, respondents in AM scored even higher than those in PC on the item “The advice stimulates me to improve my drinking behavior.” People in AM, on the other hand, stated that they found the advice more informative than people in PC. People in CP and AM agreed more with the advice than people in PC.

**Table 3:** **Standardized *T* -scores of the program evaluation items in the stage groups**

	**PC; n = 51**	**CP; n = 29**	**AM; n = 66**	**LSD contrasts**
**Questions**				
The questions were clearly stated.	50.71	48.80	49.98	PC, CP, AM
The questions were posed in logical order.	49.39	47.77	51.45	PC, CP, AM
The questions were easy to answer.	51.29	46.96	50.34	PC, CP, AM
The questionnaire was too long. ^1^	47.96	51.60	50.88	PC, CP, AM
The instructions for filling out the questionnaire were clear.	49.19	49.51	50.84	PC, CP, AM
**Advice**				
The advice was relevant for me.	48.41	52.25	50.24	PC, CP, AM
The advice was believable.	48.24	50.02	51.35	PC, CP, AM
The advice was clearly arranged.	49.19	47.84	51.58	PC, CP, AM
The advice was informative.	47.97	49.35	51.85	**PC < AM;** PC, CP; CP, AM
The advice was clear.	48.91	48.50	51.50	PC, CP, AM
The advice was complete.	50.66	47.29	50.68	PC, CP, AM
The advice helps me to improve my drinking behavior.	47.06	56.57	49.39	**PC < CP > AM;** PC, AM
The advice stimulates me to improve my drinking behavior.	45.79	58.00	49.74	**PC < CP > AM; PC < AM**
I miss information in the advice. ^1^	49.99	48.00	50.89	PC, CP, AM
The advice was consistent with my answers.	50.11	52.50	48.82	PC, CP, AM
The information was interesting.	49.39	51.01	50.03	PC, CP, AM
The information was new to me.	49.17	51.42	50.02	PC, CP, AM
The pieces of advice were too long. ^1^	50.62	51.19	49.00	PC, CP, AM
The advice was confusing. ^1^	50.62	50.46	49.32	PC, CP, AM
Too much information was given. ^1^	49.29	50.44	50.36	PC, CP, AM
I agree with the advice I received.	47.02	52.58	51.17	**PC < CP, AM**
I can learn better from video-based messages than text-based messages. ^1^	48.56	50.84	50.75	PC, CP, AM
Grade for the advice	47.61	51.96	50.98	PC, CP, AM
**Layout**				
The advice was nice in terms of layout and readability.	48.59	49.79	51.18	PC, CP, AM
I like the layout of the website.	49.24	50.23	50.49	PC, CP, AM
**Functionality**				
I had problems opening the website. ^1^	50.79	46.51	50.93	PC, CP, AM
I had problems with colors, letter types and/or pictures used in the program. ^1^	49.82	47.43	51.27	PC, CP, AM
I had problems printing the advice. ^1^	51.58	47.67	49.80	PC, CP, AM
The program is user-friendly.	48.51	52.15	50.21	PC, CP, AM
The program is a good choice for giving personal feedback.	50.24	49.89	49.86	PC, CP, AM
Grade for the program	48.02	50.35	51.37	PC, CP, AM

*Note.* PC = precontemplation; CP = contemplation/preparation; AM = action/maintenance.

CP < AM: the mean score of CP is significantly lower than that of AM.

PC, CP, AM: the mean scores of PC, CP and AM are equal.

^1^ These items are coded the other way around.

## Discussion

### Stages of change and psychosocial determinants

As a first goal, we investigated to what extent the Ø–pattern, as identified for smoking cessation, is applicable to the stage of change typology regarding intention to reduce the amount of alcohol intake.

Regarding attitude, we found as hypothesized an increase of cons in terms of alcohol between people in PC and CP, and a decrease of pros between people in CP and AM. The non-significant differences in the cons could be ascribed to a power problem, i.e. owing to a relatively small sample, significant differences may have failed to appear. The crossover of attitudes is partly in line with findings by previous studies [50,51]. Our crossover, however, seemed to occur later in the behavioral change process: between the CP stage and the AM stage. Additionally, our pros did not clearly outweigh the cons in the early stages, nor did our cons outweigh the pros in the later stages of change. In general, people in AM scored lower on pros and cons than those in PC and CP, indicating that attitude may be less important in the last stages of change. Lower scores on these attitude beliefs items are in line with the Ø–pattern.

Concerning social influence, the findings are mostly consistent with the Ø–pattern, but, owing to the division in social support and modeling, a more detailed picture can be given. Concerning social support, a relatively large difference between people in PC and CP was found, indicating that social support seems to become more prevalent in CP. The analyses confirmed that people in the AM stage had more healthy drinking role models than people in the two other groups. Thus, in order to achieve transition from one stage to another, it is recommended to increase perceptions of social support and healthy role models among those in the earlier motivational stages.

Respondents in the AM stage had higher self-efficacy expectations regarding healthy drinking in difficult situations than respondents in the PC and CP stages. This finding applies to all three kinds of self-efficacy expectations. It can be assumed that self-efficacy is strongly influenced by performance of a desired behavior [52] and people in the AM stage are by definition performing healthy drinking behavior successfully. Furthermore, people in the CP stage appeared to perceive lower social, emotional and routine self-efficacy than people in the PC stage. People in PC may make unrealistic and optimistic self-efficacy estimations. This finding is in line with a previous study [53], which found that unmotivated people often overestimate their ability to behave in a healhy manner. Another explanation for this finding may be the amount of alcohol reported. Contemplators/preparers reported drinking more than precontemplators. It can be imagined that people who drink less feel more able to abstain in certain situations.

### Stages of change and program evaluations

Regarding our second goal – a comparison of the three groups concerning their evaluation of the program – we found differences between motivational groups regarding the evaluation of the computer-tailored advice. Although all groups reported a favorable evaluation of the program, the results also suggest that respondents who were motivated to change their alcohol consumption (CP) reported more often than those who were not motivated or those in action/maintenance that the tailored advice contributed by helping and stimulating them to change their drinking behavior. These findings imply that further research is needed to analyze how the tailored advice might be adapted to increase its attractiveness and stimulating effects for people less motivated to change. Unmotivated people (PC) agreed less with the advice and perceived the advice as less informative, which may suggest that respondents in PC may need additional information targeting, for instance, awareness of their unhealthy behavior and their personal risks. The motivated group (CP) scored, for example, somewhat higher on the two items “The advice was relevant for me” and “The information in my advice was interesting,” which may indicate that these people perceived the advice as more relevant and more interesting than did individuals in precontemplation. In sum, a positive finding is that mostly no differences between the program evaluation scores between the three groups were found, suggesting that the program also addressed most items satisfactorily for unmotivated respondents.

### Study strengths and limitations

There are several reasons why the current study is important. First, previous research failed to examine differences in cognitions data across distinct stages of reducing alcohol consumption (“the Ø–pattern”). Second, this is the first study to investigate the relationship between the stages of change and the appreciation of a (computer-tailored) intervention (program evaluation). Third, in comparison with studies about the stages of change model and other health-damaging behaviors, such as smoking [52,54] and lack of physical activity [34], this study included the distinction of social influence and self-efficacy expectations according to the factor analysis when testing the Ø–pattern. The results of the factor analysis indicated that attitude could be subdivided into pros and cons, that social influence could be subdivided into support and modeling and that self-efficacy could be subdivided into social, emotional and routine self-efficacy, all consisting of reliable scales. These findings are consistent with assumptions of the I-Change model [49].

Our findings should be regarded with a certain amount of caution owing to a number of shortcomings of the study. First, the design was cross-sectional, precluding causal inferences. Longitudinal studies could give additional information about the causality between the stages of change and psychosocial determinants as well as evaluation data. Second, the sample group was small, which could have led to lack of statistical power to show significant differences, and as a result of the small sample we had to combine the different groups, based on their stage of change, into three groups. Third, the group which completed the questionnaire differed from the partial completers. Partial completers scored significantly higher on the baseline assessment of alcohol consumption (selection bias). Fourth, the Dutch alcohol consumption guideline changed in 2009. Females are now recommended to drink no more than one alcoholic drink per day and males no more than two alcoholic drinks per day [55]. In future studies, intention to comply with the new guideline should be measured. Additionally, it is recommended to have at least two alcohol-free days a week in order to prevent health problems, such as liver diseases and addiction [56]. This additional recommendation was not yet included in our feedback, but is recommended for future programs. Finally, only self-reports could be used, which may have resulted in recall difficulties, recall bias, or both. Respondents could have either overestimated or underreported their amount of alcohol intake, feelings or expectations. This can result in misclassifications [57]. Our results revealed that this was indeed the case for a small group, but reanalysis on the basis of actual consumption showed that the overall results did not change. Moreover, the possibility of participants giving socially desirable answers may have been induced by reliance on self-reports.

## Conclusions

In this study, we investigated to what extent the stages of change are relevant in the development and implementation of alcohol interventions. In most instances, these findings provide tentative support concerning essential features of the identification of stages of change. One essential feature should be that different factors are important for people in different stages [58]. Further research is needed, however, since we cannot yet say whether these factors also predict transitions from one stage to another [58]. Differences between the groups concerning the program evaluation indicate that some adjustments in terms of the alcohol intervention are desirable in order to apply it more precisely to the needs of people in different motivational stages. The intervention is positively evaluated in general, although some elements were slightly better approved by respondents motivated to change.

## Competing interests

Hein de Vries is the scientific director of Vision2Health, a company that licenses evidence-based innovative computer-tailored health communication tools. No other authors reported conflicts of interest.

## Authors’ contributions

All authors were involved in developing and implementing the program. DS significantly contributed to writing this paper, while HdV and SK were involved in revising the manuscript critically. All authors read and approved the final version of the manuscript.

## Pre-publication history

The pre-publication history for this paper can be accessed here:

http://www.biomedcentral.com/1471-2458/12/360/prepub

## Supplementary Material

Additional file 1 Factors in the proximal determinants of alcohol consumption: attitude, social influence and self-efficacy. Click here for file
