# Supplementary material for: Are the stages of change relevant for the development and implementation of a web-based tailored alcohol intervention? A cross-sectional study
Source: BMC Public Health. 2012 May 17;12:360. doi: 10.1186/1471-2458-12-360 (PMC3487757; doi:10.1186/1471-2458-12-360)
Supplement: Additional file 1 — Factors in the proximal determinants of alcohol consumption: attitude, social influence and self-efficacy. [file 1471-2458-12-360-S1.pdf]

## Additional file 1 - Factors in the proximal determinants of alcohol consumption: attitude, social influence and self-efficacy

| **Items** | **Factors** | |
|---|---|---|
| **Attitude (-2, +2)**<br><br>*When I drink alcohol, …* | **Pros of alcohol**[a]<br>(α = .93) | **Cons of alcohol**[b]<br>(α = .95) |
| *… I feel more confident.* | .80 | |
| *… I feel more confident when I am in company.* | .77 | |
| *... I feel happier.* | .76 | |
| *… it makes me funny.* | .73 | |
| *… it makes it easier for me to approach others.* | .72 | |
| *… it makes me loosen up.* | .71 | |
| *… I fit in better.* | .70 | |
| *… I get more attention from my friends.* | .70 | |
| *… I am not bored.* | .69 | |
| *… I can cope better with problems.* | .68 | |
| *… I feel more relaxed.* | .67 | |
| *… I have a lot of friends.* | .66 | |
| *… people look up to me.* | .65 | |
| *… I feel like somebody.* | .62 | |
| *… it is exciting.* | .60 | |
| *… I relax.* | .60 | |
| *… I do not feel lonely.* | .57 | |
| *… it helps with feelings of frustration, depression and/or stress.* | .53 | |
| *… my concentration improves.* | .49 | |
| *… it is a pleasant/cosy feeling.* | .49 | |
| *… I can more easily fall asleep.* | .41 | |
| *… it makes me cause conflicts with others.* | | .87 |
| *… I have problems.* | | .84 |
| *… I get in conflict with others.* | | .82 |
| *… the relationship with my partner becomes worse.* | | .81 |
| *… I feel ashamed.* | | .81 |
| *… I more often do things that I regret.* | | .80 |
| *… my condition gets worse.* | | .77 |
| *… I have problems at work.* | | .77 |
| *… I make mistakes.* | | .73 |
| *… it is bad for my brain.* | | .71 |
| *… the risk of developing cancer increases.* | | .70 |
| *… I become aggressive.* | | .69 |
| *… I cause traffic accidents.* | | .68 |
| *… it impairs/decreases my sexual functioning.* | | .67 |
| *… I become emotional.* | | .67 |
| *… it is bad for my health.* | | .66 |
| *… I do not feel fit/well.* | | .66 |
| *… the risk of developing cardiovascular diseases increases.* | | .65 |
| *… it is bad for my liver.* | | .65 |
| *… I get tired.* | | .59 |
| *… the hangover is a disadvantage.* | | .58 |
| *… I become more stupid.* | | .54 |
| *… I do not sleep well.* | | .50 |
| *… it costs a lot.* | | .44 |

| **Social influence (-2, +2)** | **Modeling** [c] (α = .70) | **Support** [d] (α = .85) | |
|---|---|---|---|
| *My partner does not drink more than two glasses* of alcohol per day.* | *.88* | | |
| *My family do not drink more than two glasses* of alcohol per day.* | *.83* | | |
| *My friends do not drink more than two glasses* of alcohol per day.* | *.66* | | |
| *My colleagues do not drink more than two glasses* of alcohol per day.* | *.51* | | |
| *My partner encourages me not to drink more than two glasses* of alcohol per day.* | | *.88* | |
| *My family encourage me not to drink more than two glasses* of alcohol per day.* | | *.88* | |
| *My friends encourage me not to drink more than two glasses* of alcohol per day.* | | *.84* | |
| *My colleagues encourage me not to drink more than two glasses* of alcohol per day.* | | *.76* | |
| **Self-efficacy**<br>*I am able to drink no more than two glasses* of alcohol …* | **Social SE** [e]<br>(α = .94) | **Emotional SE** [e]<br>(α = .97) | **Routine SE** [e]<br>(α = .88) |
| *… when someone offers me a drink.* | *.92* | | |
| *… when there is something to celebrate.* | *.91* | | |
| *… when I am at a party.* | *.90* | | |
| *… when I am at a café or restaurant.* | *.83* | | |
| *… when I see people around me drinking alcohol.* | *.78* | | |
| *… when I see somebody else enjoying a drink.* | *.61* | | |
| *… when others criticize me when I do not want to drink.* | *.55* | | |
| *… when I experience feelings of depression.* | | *.98* | |
| *… when I am sad.* | | *.97* | |
| *… when I feel stressed or nervous.* | | *.94* | |
| *… when I have problems.* | | *.93* | |
| *… when I am in a bad mood.* | | *.91* | |
| *… when I am annoyed, angry or frustrated.* | | *.87* | |
| *… when I cannot fall asleep.* | | *.79* | |
| *… when I am around others and feel uncomfortable.* | | *.74* | |
| *… when I cannot concentrate.* | | *.67* | |
| *… when I am bored.* | | *.67* | |
| *… when I get up in the morning.* | | | *.98* |
| *… when I have a break.* | | | *.94* |
| *… after doing sports.* | | | *.63* |
| *… during lunch or dinner.* | | | *.52* |
| *… when I see or smell alcohol.* | | | *.46* |

*Note.* *This item refers to females (three glasses for males). [a]+2 = *positive attitude*, -2 = *negative attitude;* [b]+2 = *negative attitude*, -2 = *positive attitude;* [c]+2 = *positive modeling*, -2 = *negative modeling*; [d]+2 = *high social support*, -2 = *low social support;* [e]+2 = *high self-efficacy*, -2 = *low self-efficacy*
